# Nitric oxide-releasing emulsion with hyaluronic acid and vitamin E†

**DOI:** 10.1039/c9ra03840j

**Published:** 2019-07-15

**Authors:** Janet P. Yapor, Jenna L. Gordon, Christina N. Henderson, Melissa M. Reynolds

**Affiliations:** Department of Chemistry, Colorado State University Fort Collins CO 80523 USA Melissa.Reynolds@colostate.edu; Department of Biochemistry and Molecular Biology, Colorado State University Fort Collins CO 80523 USA; School of Biomedical Engineering, Colorado State University Fort Collins CO 80523 USA

## Abstract

*S*-Nitrosoglutathione (GSNO) is a naturally available *S*-nitrosothiol that can be incorporated into non-toxic formulations intended for topical use. The value of nitric oxide (NO) delivered topically relates to its well-studied physiological functions such as vasodilation, angiogenesis, cell proliferation and broad-spectrum antibacterial activity. Previously reported topical NO-releasing substrates include polymeric materials that exhibit non-toxic behaviors on dermal tissue such as polyethylene glycol. However, they do not serve as humectants nor provide vitamins to the skin. In this study, GSNO was added to an emulsion that was fortified with α-tocopheryl acetate (vitamin E) and hyaluronic acid. The average total NO content for the NO-releasing emulsion was 58 ± 8 μmol g^−1^ at 150 °C and the cumulative NO release over 53 h at physiological temperature (37.4 °C) was 46 ± 4 μmol g^−1^. The GSNO concentration in the lotion was optimized in order to reach a pH value similar to that of human skin (pH 5.5). The viscosity was analyzed using a rotational viscometer for the *S*-nitrosated and the non-nitrosated emulsions to obtain a material that can be readily spread on dermal tissue. The viscosity values obtained ranged from 7.88 ± 0.99 to 8.50 ± 0.36 Pa s. Previous studies have determined that the viscosity maximum for lotions is 100 Pa s. A low viscosity increases the diffusion coefficient of active ingredients to the skin given that they are inversely proportional as described by the Einstein–Smoluchowski equation. The effect of the *S*-nitrosated and non-nitrosated emulsions on adult human dermal fibroblasts (HDFs) was assessed in comparison to untreated HDFs using Colorimetric Cell Viability Kit I-WST-8. The findings indicate that neither the *S*-nitrosated nor non-nitrosated emulsions induced cytotoxicity in HDFs.

## Abstract 2

*S*-Nitrosoglutathione (GSNO) is a naturally available *S*-nitrosothiol that can be incorporated into non-toxic formulations intended for topical use.

## Graphical abstract

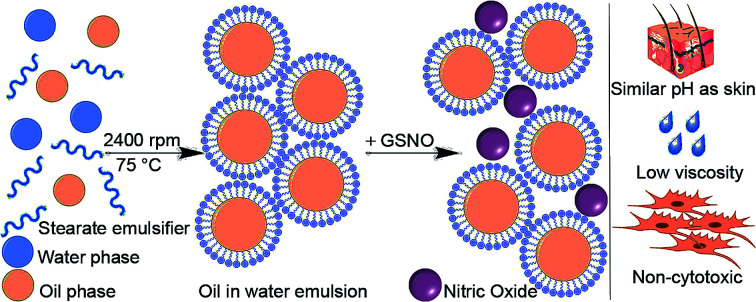

## 1. Introduction

Nitric oxide (NO) is an endogenous molecule involved in various physiological processes including vasodilation,^1,2^ angiogenesis,^3^ inhibition of platelet activation,^4,5^ and broad-spectrum antibacterial activity as part of the immune response.^6^ The beneficial effects of NO at various concentrations have inspired the creation of NO-releasing platforms for a wide range of therapeutic uses.^7,8^ For example, the vasoactivity and antithrombogenicity of NO can be harnessed to prepare topical therapeutics that promote perfusion, leading to increased oxygenation of dermal tissue and improved clinical outcomes.^9,10^ In such formulations, exogenous NO diffuses through the epidermis and enters the local vasculature, where it can enhance blood flow and exert other beneficial effects through various biochemical pathways.^11–13^ Because NO is a highly reactive gas with a short half-life, storage and delivery is often achieved through use of NO donor molecules that exhibit greater stability. Common NO donors include *S*-nitrosothiols (RSNOs), *N*-diazeniumdiolates (NONOates), and inorganic nitrite.^7,14–16^ Formulations incorporating these NO donors have demonstrated considerable promise in topical applications.

In a murine model, wound healing was dramatically influenced by exposure to a cream containing sodium nitrite and citric acid.^17^ Following the initial coagulation stage, the rate and extent of wound healing in both normal and diabetic mice was enhanced by topical NO release. Seabra *et al.* reported the preparation of NO-releasing hydrogels from addition of *S*-nitrosoglutathione (GSNO) or *S*-nitroso-*N*-acetylcysteine to the commercial polyalkylene oxide triblock copolymer Synperonic F-127.^18^ These NO-releasing hydrogels were found to elevate blood flow when monitored by laser Doppler flowmetry. Dermal microdialysis revealed that dermal nitrite concentration (an indicator of NO metabolism) was generally directly correlated with blood flow. In a subsequent study, topical use of an NO-releasing polyester derived from ethylene glycol and *S*-nitrosomercaptosuccinic acid was explored.^19^ When this material was applied to the skin of human subjects in ambient light, NO release induced local vasodilation and hyperaemia. The authors proposed that this effect may facilitate wound healing applications, in addition to other possible uses. The antibacterial properties of NO can also be exploited topically, as demonstrated by the successful treatment of acne vulgaris with a gel containing the NO donor NVN1000, a polysiloxane-based NONOate.^11^ Previous studies have reported a polysaccharide derivative that released a total of 49.5 ± 5.0 μmol g^−1^ over the course of 24 h, with efficacious antibacterial action against *Escherichia coli*, *Acinetobacter baumannii* and *Staphylococcus aureus*. The report confirmed 8-log reduction of bacteria colony forming units for all bacterial strains under evaluation.^20^ A different report that investigated the extent of cell proliferation on cardiovascular-related cells confirmed that the NO donor concentrations that allowed cells to proliferate was in the range of 2–20 μmol L^−1^.^21^ Such NO release was able to delay the differentiation of embryonic cells and promote their survival. A different study using *S*-nitroso-*N*-acetylpenicillamine (SNAP) concluded that a NO donor concentration of 10–50 μmol L^−1^ was beneficial for promoting proliferation and cell survival of bone marrow stromal cells.^22^

It is evident that NO-releasing topical formulations have substantial value in the treatment of wounds, hemodynamic disorders, and bacterial infection. In particular, inclusion of an NO donor within a viscous emulsion would facilitate topical application and permit combination with other therapeutic additives. Herein, an emulsion with non-toxic components is reported that incorporates *S*-nitrosoglutathione (GSNO), a naturally-occurring RSNO, as well as α-tocopheryl and hyaluronic acid. The GSNO concentration was optimized in order to achieve physiologically relevant NO release that could promote vasodilation and cell proliferation.^23,24^ α-Tocopheryl acetate, the second additive, is a naturally-occurring compound in the skin that is obtained through dietary sources.^25^ Topical application of α-tocopheryl provides a potent, localized delivery of the antioxidant that is unachievable through the diet alone.^26^ Studies have shown that topical vitamin E plays a role in photoprotection,^27^ serves as an anti-inflammatory agent,^28^ promotes the healing of wounds,^29^ and supplements moisturizing effects.^30^ Hyaluronic acid, another naturally occurring biomolecule found in all organs of the human body, was included as a third additive.^31^ Aging, due to both intrinsic and extrinsic factors, affects the overall ability of the skin to retain moisture and produce its own hyaluronic acid.^32^ Evidence suggests that hyaluronic acid delivery through injections and formulations is a valuable solution for retaining moisture within the skin.^33^

**Fig. 1:**
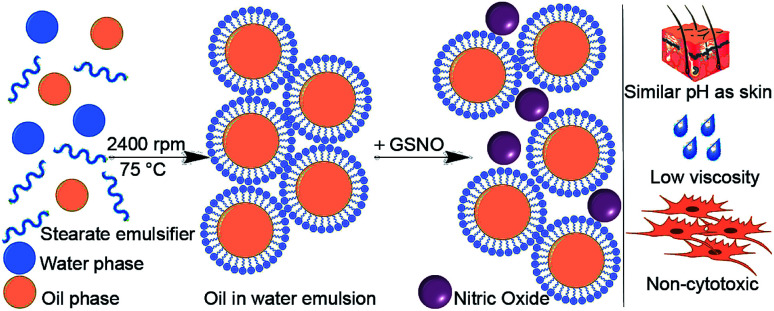
Illustrated scheme of the emulsion formulation.

Several analyses were performed on the formulated emulsion to demonstrate applicability. First, viscosity was analyzed to obtain an appropriate consistency for a sample that could be applied locally, eliminating disturbances to the surrounding tissue. Additionally, the pH was measured over 12 weeks to investigate the stability of the *S*-nitrosated emulsion. Finally, cell studies were performed using HDFs to assess cytotoxicity of the *S*-nitrosated and non-nitrosated emulsions. The formulation is the first to report the combination of nitric oxide, vitamin E, and hyaluronic acid in an emulsion intended for topical use. The results of cell proliferation studies and characterization techniques are discussed herein. The combination of beneficial additives that allow moisture retention by dermal tissue, in combination with NO-release make this system a relevant platform with potential future applications in wound healing. A scheme that represents the formulation of the emulsion is presented in Fig. 1.

## 2. Materials and methods

### 2.1 Materials

dl-α-Tocopheryl acetate (Vitamin E; 97%), *n*-propyl-4-hydroxybenzoate (99%), methyl 4-hydroxybenzoate (99%), 1-hexadecanol (98%), isopropyl tetradecanoate (98%), and triethanolamine (>98%) were purchased from Alfa Aesar (Ward Hill, MA, USA). Hyaluronic acid sodium salt from *Streptococcus equi* (mol. wt ∼1.5–1.8 × 10^6^ Da, ≤1% protein) and mineral oil (light) were procured from Sigma Aldrich (St. Louis, MO, USA). Glycerol was purchased from Mallinckrodt (Phillipsburg, NJ, USA). Stearic acid (>98%) was obtained from TCI (Tokyo, Japan). Glyceryl stearate SE (self emulsifying: glyceryl monostearate with 3–6% potassium stearate) and carbomer 980 QD were procured from MakingCosmetics Inc (Snoqualmie, WA, USA). Reduced glutathione (GSH) was purchased from VWR International (Radnor, PA, USA). Hydrochloric acid (HCl) and EPA vials were obtained from Thermo Fisher Scientific (Waltham, MA, USA). Sodium nitrite (NaNO_2_) was purchased from EMD Millipore (Burlington, MA, USA). Ultrapure water (18.2 MΩ cm) was supplied by a Millipore Direct-Q water purification system (EMD Millipore). Adult human dermal fibroblasts (lot # 80616174) were purchased and maintained with fibroblast basal medium (FBM) (lot # 80921174) supplemented with fibroblast growth kit – low serum (lot # 80712184) and penicillin–streptomycin–amphotericin B (lot # 80118182) purchased from American Type Culture Collection (Manassas, VA, USA). Cell Viability Kit I-WST 8 (lot # 443M374) was purchased from Promega (Madison, WI, USA). Trypan Blue Solution (lot #RNBD1973) was purchased from Sigma-Aldrich. Trypsin/EDTA Solution for Primary Cells (lot # 80623180) and Trypsin Neutralizing Solution (lot# 80002512) were purchased from American Type Culture Collection (Manassas, VA, USA).

### 2.2 Characterization techniques

UV-Vis absorption studies were performed using a Nicolet Evolution 300 UV-Vis spectrophotometer (Thermo Electron Corporation, Madison, WI, USA). Diffuse reflectance UV-Vis (DR UV-Vis) spectra were acquired using a Praying Mantis accessory (Harrick Scientific Products, Inc., Pleasantville, NY, USA) and a Spectralon® baseline. Over the course of 12 weeks, pH values were obtained using a Mettler Toledo Seven Easy pH meter equipped with a Mettler Toledo InLab® Routine Pro pH probe (Mettler Toledo, Columbus, OH, USA). Dilutions for pH tests were prepared using ultrapure water to make 11.1% w/v solutions at room temperature. Viscosity was analyzed using a Fungilab Viscolead Pro rotational viscometer (Fungilab, Barcelona, Spain). Viscosity measurements were obtained for *S*-nitrosated and non-nitrosated, hyaluronic acid-free samples using spindles L4 Fungilab at 60 revolutions per min (rpm) and L3 Fungilab at 12 rpm, respectively. The sample temperature was controlled with a water bath and maintained at 25 °C.

#### Chemiluminescence-based NO analysis

NO release from *S*-nitrosated emulsions was evaluated using Sievers chemiluminescence NO analyzers (NOA 280i; Zysense, Weddington, NC, USA) following our previously reported procedure.^34^ The instruments were calibrated prior to each analysis using nitrogen as the zero gas and 43.6 ppm NO/nitrogen as the calibration gas. The nitrogen sweep gas flow during the analysis was maintained at 200 mL min^−1^. Total NO content was obtained by heating GSNO emulsions (*n* = 3; 9 mg) and appropriate controls at 150 °C in the absence of solvent followed by irradiation of light at 365 nm. This promoted the thermal and photodecomposition of the RSNO groups. The NO emission from this process was used to quantify the thermally and photo-releasable NO present in each material. In addition, NO release at physiological temperature was determined by heating GSNO emulsion samples (*n* = 3) in the absence of solvent at 37 °C. NO release was measured for 53 h in vessels protected from direct exposure to light to prevent further photodecomposition of the RSNO. Measurements were performed in triplicate and the average and standard deviation reported.

### 2.3 Synthesis of materials

#### Emulsion preparation

The emulsion was prepared from vigorous stirring of aqueous and lipophilic phases. For the aqueous phase, a 2% w/v solution of carbomer 980 QD was prepared in ultrapure water at room temperature 24 h prior to the emulsification. The following day, ultrapure water was heated to 75 °C and 79.7 mL was added to glycerol (3 g), triethanolamine (0.9 g), methyl 4-hydroxybenzoate (0.1 g), and carbomer 980 QD 2% w/v in ultrapure water (5 g). The aqueous phase was maintained at 75 °C in a cylindrical glass vessel with dimensions 6 cm × 19 cm (diameter × height). The lipophilic phase consisted of 1-hexadecanol (2 g), stearic acid (0.8 g), glyceryl stearate SE (1.5 g), *n*-propyl-4-hydroxybenzoate (0.05 g), isopropyl tetradecanoate (0.85 g), α-tocopheryl acetate (0.995 g) and mineral oil (0.85 g), which were heated to 75 °C until melted. The lipophilic phase was slowly added to the aqueous phase and stirred at 2400 rpm for 30 min at 75 °C. Emulsification was achieved using a IKA® RW 20 digital mechanical stirrer (IKA Works, Inc., Wilmington, NC, USA). The white emulsion was transferred to a glass container with a cap and stored at room temperature shielded from direct exposure to light.

#### Synthesis and characterization of *S*-nitrosoglutathione

*S*-Nitrosoglutathione (GSNO), the NO donor in this study, was synthesized based on a previously reported method.^34^ Briefly, a mixture of 5 mmol of reduced glutathione (GSH), 8 mL of cold Millipore water, and 2.5 mL of 2 M hydrochloric acid (HCl) was stirred over ice for 10 min, followed by adding 5 mmol of sodium nitrite (NaNO_2_) into this solution to initiate *S*-nitrosation. The mixture was stirred further for 40 min over ice before the addition of 10 mL of cold acetone. The resulting GSNO was collected and washed with 10 mL of cold water and 10 mL of cold acetone before being dried under vacuum for 4 h. The purity of GSNO was characterized using UV-Vis spectrophotometric techniques by measuring the absorbance at 335 nm to ensure >95% purity.^35^ The GSNO was stored in an amber EPA vial at −20 °C before use to prevent both photolytic and thermal decomposition.

#### Addition of GSNO to emulsion

Hyaluronic acid sodium salt (50 mg) was added to the emulsion (5 g) to obtain a 1% w/w mixture in an amber vial. The mixture was vortexed briefly to incorporate all solids, then stirred at 725 rpm overnight at room temperature. The process was followed by the addition of GSNO (88.4 mg) to obtain a 1.72% w/w concentration. The mixture was alternately vortexed for 2 min and stirred at 1100 rpm for 4 min over the course of 1 h. The samples were stored at 4 °C until further use.

### 2.4 Cell studies

#### Cell culture

HDFs were grown in fibroblast basal medium supplemented with fibroblast growth kit – low serum and penicillin–streptomycin–amphotericin B solution at 37 °C in a humidified incubator with 5% CO_2_. At ∼90% confluence, monolayer HDF were detached using trypsin/EDTA solution for primary cells, neutralized with trypsin neutralizing solution, centrifuged and resuspended. Cells were counted using the trypan blue exclusion method and seeded at a density of 100 000 cells per mL in a 96-well plate.

#### Cell viability assays

The effect of the *S*-nitrosated and non-nitrosated emulsions on HDFs was assessed using Colorimetric Cell Viability Kit I-WST-8 after 24 h of exposure. Cells were counted prior to seeding at a density of 100 000 cells per mL in a 96-well plate. After 24 h, the FBM was aspirated and replaced with one of three solutions: FBM as a control (9 samples), *S*-nitrosated emulsion (50 mg) dissolved in FBM (3 replicates × 9 samples), or non-nitrosated (50 mg) emulsion dissolved in FBM (3 replicates × 9 samples). Each sample was plated in a separate row in 9 consecutive wells. After 24 h, each solution was aspirated and replaced with FBM once again. At this point, cell viability was assessed with Colorimetric Cell Viability Kit I-WST-8 to determine the effect of each emulsion on HDFs in comparison to untreated HDFs. The absorbance value of all samples was measured at 450 nm using a BioTek Synergy 2 Multi-Detection Microplate Reader. An average and standard deviation of the 9 untreated HDF samples was determined and compared to the absorbance value of each of measured sample. These values were averaged and the standard deviation of each set of 27 treated samples was calculated. ANOVA and the Student's *t*-test were performed to determine the statistical significance of the measured data.

## 3. Results and discussion

The emulsion was prepared using commercially available ingredients that are common in cosmetic formulations. The emulsion provides a carrier medium for GSNO, which acts as an NO donor. GSNO was selected due to its stability and natural abundance in the body as a simple tripeptide of glycine, cysteine and glutamic acid. The aqueous phase of the emulsion was prepared with ultrapure water. The primary thickener was carbomer 980 QD, a pentaerythritol-crosslinked poly(acrylic acid) (PAA) formulation. Carbomer 980 was added to the aqueous phase at a concentration of 2% w/v to prevent phase separation and stabilize the emulsion. Glycerol was included as a humectant that functions to attract water and draw additional moisture into the epidermis,^36^ while methyl 4-hydroxybenzoate was added as a preservative. Triethanolamine was used as a neutralizer in order to balance the pH. The lipophilic phase was composed of 1-hexadecanol, stearic acid, glyceryl stearate SE, *n*-propyl-4-hydroxybenzoate, isopropyl tetradecanoate, and mineral oil. Stearic acid and glyceryl stearate SE are common emulsifiers. 1-Hexadecanol is a coemulsifier that increased the stability of the emulsion. The preservative for the lipophilic phase utilized *n*-propyl-4-hydroxybenzoate. This phase of the lotion moisturizes because of the added occlusive agents isopropyl tetradecanoate and mineral oil. Furthermore, occlusive compounds form a physical layer atop the skin and prevent the loss of water.^36^ Vitamin E was a component of interest given that it is a biologically available antioxidant. In particular, α-tocopheryl acetate was used, as opposed to the other forms of vitamin E, given that it is the only form that is reported to meet human requirements.^37^ Hyaluronic acid was included in the formulation due to its natural occurrence and its ability to regulate moisture within cells.

### 3.1 Kinematic viscosity analysis

**Fig. 2:**
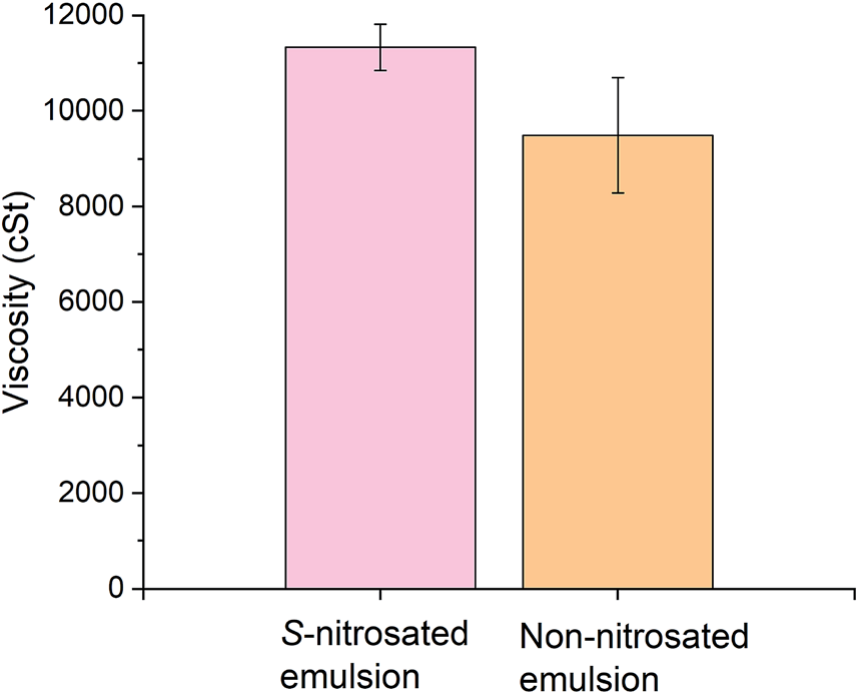
Kinematic viscosity of *S*-nitrosated and non-nitrosated emulsion samples. All samples were tested in replicate (*n* ≥ 3) and the results are displayed as the mean ± standard deviation.

**Fig. 3:**
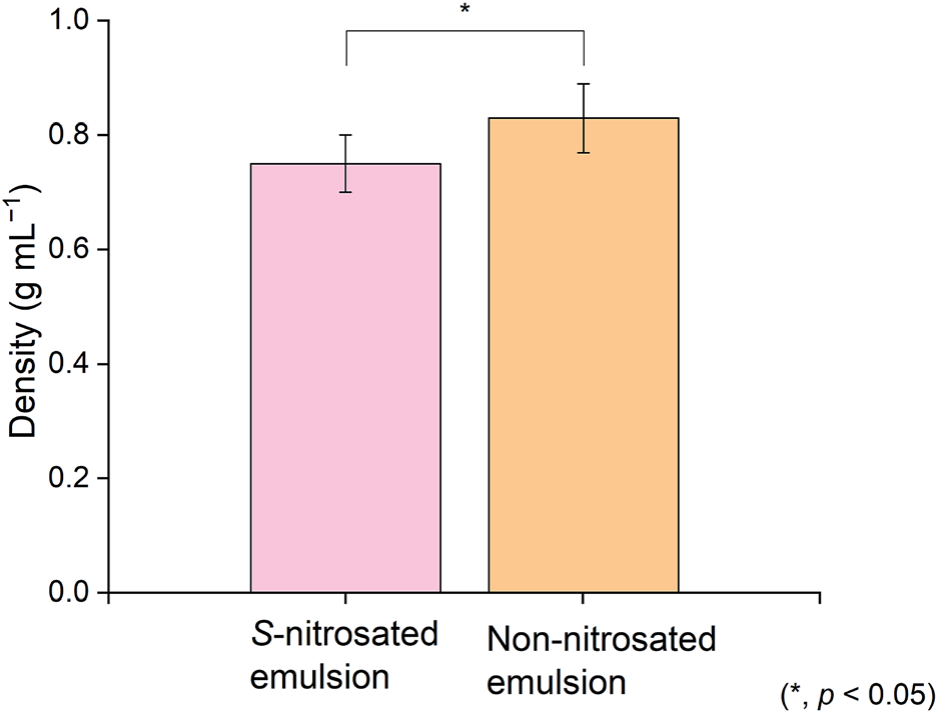
Density of *S*-nitrosated and non-nitrosated emulsion samples. All samples were tested in replicate (*n* ≥ 3) and the results are reported as the mean ± standard deviation.

Kinematic viscosity analysis was performed to establish a viscosity range that would permit retention of the emulsion on the skin. The GSNO emulsion and a control were evaluated without dilution at a constant temperature of 25 °C. Fig. 2 and 3 present the kinematic viscosity and density values obtained for each material. Compared to common liquids, the viscosity of the emulsion is similar to that of molasses (10 000 cSt). Although there was no statistically significant difference in viscosity between the GSNO emulsion and a control lacking GSNO and hyaluronic acid, the density decreased with inclusion of these components (Table S1†).

The physicochemical properties of dermal products determine their viscosity (*η*), and modulate the magnitude of diffusion coefficient (*D*) as expressed by the Einstein–Smoluchowski equation (*D* = *kT*/6π*rη*). Where, *k* is the Boltzman constant, *T* represents the temperature in kelvin and *r* is the observed radius of the active ingredient particle.^38^ Flux across the skin can be enhanced by an increase in the diffusion coefficient, partition coefficient and/or the concentration of the permeant in the vehicle. The continuous change in equilibrium between the vehicle, the active ingredient, and the skin all contribute to the flux of active ingredients across the skin.^39^ The vehicle in this study was the emulsion, the active ingredients were nitric oxide, vitamin E and hyaluronic acid. In addition, skin penetration of an active ingredient may be optimized by a careful selection of the vehicle.^40^ Furthermore, it should be taken into consideration that the vehicle will change once it is applied to the skin.^41^ The benefit of having an emulsion with low viscosity comes primarily from the constitution of the emulsion, which was approximately 80% w/w water. Consequently, water evaporation permits the diffusion process given that the active ingredients can diffuse through the skin as the vehicle evaporates.^42^

Guidelines that enhance the flux of active ingredients include: maximum thermodynamic activity of the permeant in the vehicle, supersaturation, and the use of penetration enhancers that increase diffusivity across the skin. The main constituent of the emulsion, water, has been studied for its ability to modify skin penetration.^43^ An increase in water content usually results in increased transdermal delivery of hydrophilic and lipophilic permeants.^44^ The kinematic viscosity calculated was 9501 ± 1203 cSt for the non-nitrosated emulsion, and was 11 330 ± 485 cSt for the *S*-nitrosated sample. Using the density of the emulsions presented in Table S1,† the viscosity can be converted to Pa s for comparison. The viscosity can then be expressed as 7.88 ± 0.99 Pa s for the non-nitrosated emulsion, and 8.50 ± 0.36 Pa s for the *S*-nitrosated emulsion. Previous studies have determined that the viscosity maximum for lotions is in the order of magnitude of *η*_max_ = 100 Pa s and for creams *η*_max_ = 1000 Pa s.^45^ In addition, reports have indicated that the viscosity of body lotion is 10.55 Pa s and that for skin cream is 44.78 Pa s.^46,47^ The results are comparable to the data reported herein. Hence, the viscosity values obtained in this study suggest that the emulsions are suitable for the intended application.

### 3.2 Analysis of pH

The pH of healthy skin has been reported to fall within the range of 4.0–7.0, but rigorous studies suggest that normal skin pH is slightly below 5. In addition, it has been demonstrated that a pH between 4 and 5 is optimal for maximum absorption of external compositions into the skin.^48^ Furthermore, natural skin surface is on average below 5, which is beneficial for its resident flora.^49^ The pH of the emulsion with α-tocopheryl acetate, without hyaluronic acid, was measured at different GSNO concentrations including, 1.0%, 1.5%, 2.0%, 2.5%, 3.0% and 6.0% w/w, with resulting pH values of 7.43, 6.75, 5.36, 4.37, 3.88, and 3.24, respectively (Fig. S3†). HA was not included in the initial studies since the addition did not affect the pH of the emulsion.

The emulsion was unstable when the GSNO concentration was greater than 3.0% w/w, an outcome that is attributable to increased acidity of the emulsion. It was experimentally determined that the emulsion was stable at pH ranges from 7.43 to 4.37 and unstable when the pH was lower than 3.88. The emulsion was unstable when the GSNO concentration was greater than 3.0% w/w, an outcome that is attributable to increased acidity of the emulsion. It was experimentally determined that the emulsion was stable at pH ranges from 7.43 to 4.37 and unstable when the pH was lower than 3.88. This lack of emulsion stability is likely due to protonation of stearate under acidic conditions. The oil in water emulsion was achieved due to the reduced interfacial tension between the lipophilic and hydrophilic phases. The anionic end of the emulsifier is able to stabilize the emulsion only if the emulsifier remains deprotonated, a state that is disfavored at low pH.

The pH was measured in triplicate over the course of 12 weeks for *S*-nitrosated emulsion with α-tocopheryl acetate and hyaluronic acid. Dilutions of the emulsion were made using ultrapure water to obtain a final concentration of 11.1% w/v. Samples were stirred vigorously prior to pH tests. The results obtained from the analyses are shown in Table S2.† The gradual decrease in pH is attributable to decomposition of GSNO and concomitant release of NO, which may react with water and oxygen to form acidic products such as nitrous (HNO_2_) or nitric acid (HNO_3_).

**Fig. 4:**
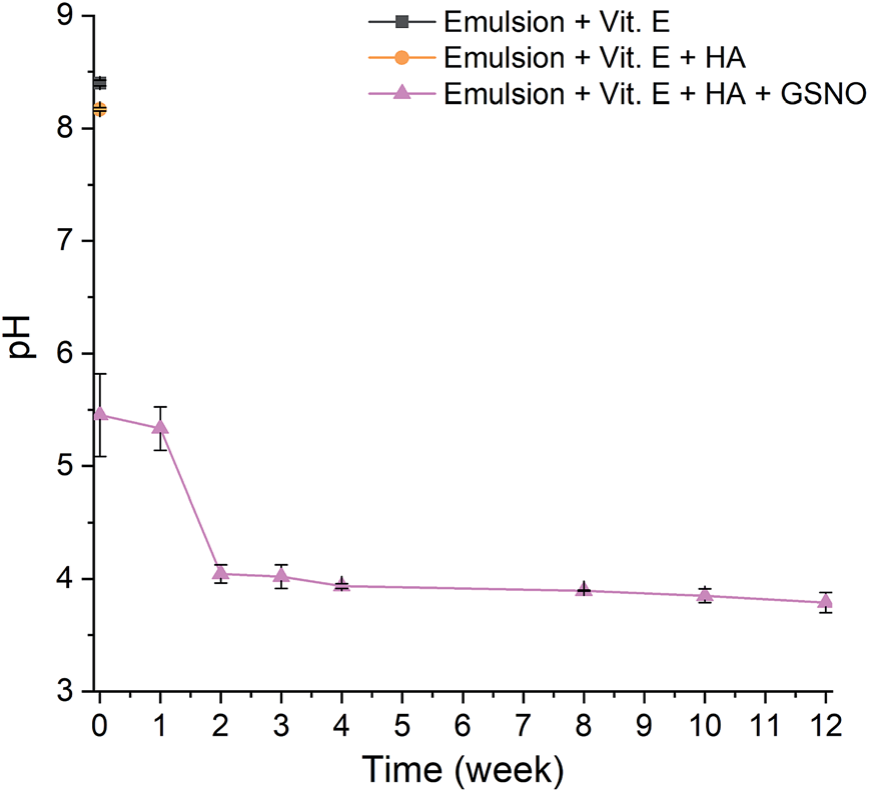
Analysis of pH of the emulsion with various additives. Each data point represents an average of *n* ≥ 3 replicates and the standard deviation. The labels indicate the composition of each sample, where α-tocopheryl acetate (vitamin E) (Vit. E) and hyaluronic acid (HA) are abbreviated accordingly.

These results demonstrate that the pH of the emulsion remains within the specified range over the course of 12 weeks, making it suitable for application to the skin (Fig. 4).

### 3.3 Chemiluminescence-based NO analysis

Previous reports have demonstrated that different physiological responses are observed at particular instantaneous NO concentrations. Mediation of proliferative and protective effects occurs at <1–30 nM, followed by apoptosis protection in the range of 30–60 nM, and tissue injury protection at 100 nM.^50^ The desired local NO concentration for tissue regeneration is therefore in the range of 1–100 nM. At a concentration of 1.72% w/w, the NO release calculated for 53 h at physiological temperature was 46 ± 4 μmol g^−1^ and the total NO content was 58 ± 8 μmol g^−1^. These values are provided in Table 1 and were collected from NO release experiments performed using 3 different batches of emulsion, to which α-tocopheryl acetate and hyaluronic acid were added, followed by the addition of GSNO. The formulations were the same in all three batches for both, cumulative NO release and total NO content experiments. Determination of total NO load was performed by heating the emulsion to 150 °C, the thermal decomposition temperature of GSNO. This procedure was followed by irradiation with light (365 nm) to promote photodecomposition of any remaining RSNO groups. Total NO load can be used to compare how much NO is present in the emulsion compared to the quantity of NO released at physiological temperature. The percent NO release is 79.3% considering that the experiment performed at 150 °C provided the total NO loaded. In order to quantify the total NO loading, the experiment was allowed to proceed for 2 h. At physiological temperature (37.4 °C) the NO release was monitored for 53 h. The theoretical availability of GSNO in a solution of 1.72% GSNO is 52 μmol g^−1^, which is comparable to the results obtained for total NO content. Previous reports have shown similar results in terms of NO release such as 45 μmol mL^−1^ over 24 h.^19^ Furthermore, the similar NO loading results in physiological effects that would be relevant for tissue regeneration, such as tissue injury protection. The advantage of having a longer release profile that lasts 53 h with a low NO release is that NO can continue reacting on the skin without causing apoptotic effects, which are typically associated with NO concentrations higher than 1 μM.

**Table 1:** Summarized NO content data^a^

	Cumulative NO Release (μmol g^−1^)	Total NO load^b^ (μmol g^−1^)
*S*-Nitrosated emulsion	46 ± 4	58 ± 8

a Release measured at physiological temperature (37 °C) for 53 h.

b Values determined by NO analysis through thermal decomposition of the RSNO at 150 °C. All samples were tested in replicate (*n* ≥ 3) and the results are reported as the mean ± standard deviation.

**Fig. 5:**
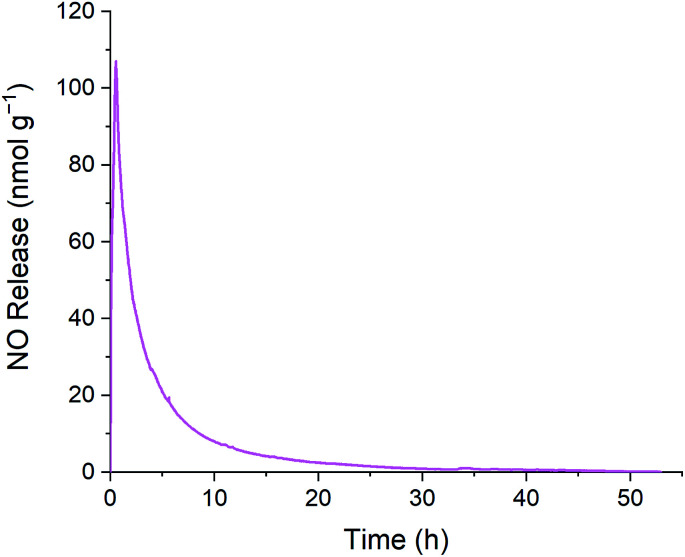
Representative NO release profile of *S*-nitrosated emulsion at physiological temperature.

**Fig. 6:**
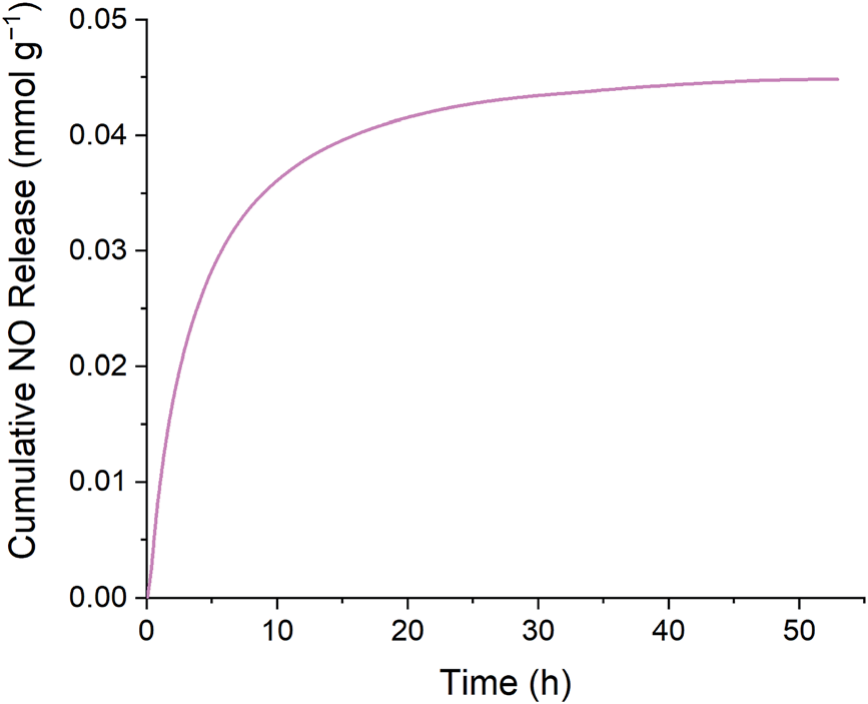
Representative cumulative NO release data of *S*-nitrosated emulsion at physiological temperature.

The release profile depicted in Fig. 5 reflects exponential decay behavior that is commonly observed during decomposition of RSNOs. The cumulative release plot shown in Fig. 6 gradually plateaus as the supply of releasable NO is exhausted. The higher initial NO release during the first few hours of the experiment may be beneficial from an antibacterial perspective. The initial burst of NO release results from the chemistry of RSNOs, which are often observed to produce NO through exponential decay. Heat, light, and the presence of certain transition metal ions are generally understood to promote cleavage of gaseous NO from RSNOs.^35^ To produce the profile shown in Fig. 5 the emulsion was placed in a glass vessel and heat was introduced to the system by increasing the temperature to 37.4 °C. This heating process causes GSNO to undergo NO-forming decay according to the kinetics illustrated in Fig. 6. The slower NO release observed afterward may provide NO at a level that supports cell proliferation, while avoiding cell apoptosis.^24,51^

Using the density of the *S*-nitrosated emulsion and its instantaneous NO release, it can be calculated that at the later stages of NO release, when the signal has plateaued after 49 h, the instantaneous NO release is 123 pmol g^−1^, which is equivalent to 92.3 nM. At 53 h the instantaneous release is 53.7 pmol g^−1^, which is equivalent to 40 nM. As described previously, this NO release is in the target range for tissue injury protection between 1–100 nM.^50^

**Fig. 7:**
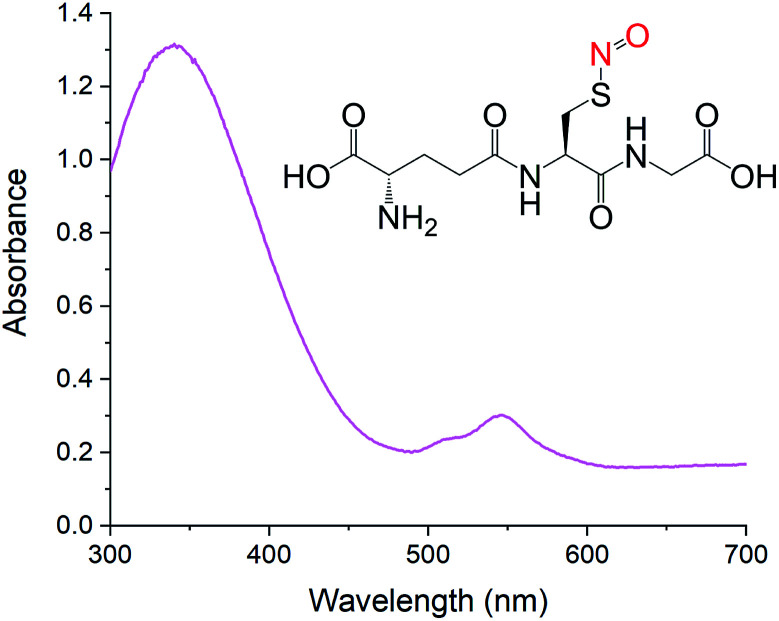
DR UV-Vis spectrum of *S*-nitrosated emulsion with characteristic RSNO features. The structure of GSNO is shown as an inset.

The continuing presence of GSNO was confirmed by UV-Vis spectrophotometry using diffuse reflectance. The method was chosen given the limited solubility of the sample in most solvents. Fig. 7 shows a representative DR UV-Vis spectrum of the *S*-nitrosated emulsion. Characteristic RSNO absorption peaks were observed at 339 ± 1.5 (π → π*) and 546 ± 0.6 nm (n_N_ → π*). All samples were tested in replicate (*n* ≥ 3) and the results are reported as the mean ± standard deviation.

### 3.4 Cell viability assays

**Fig. 8:**
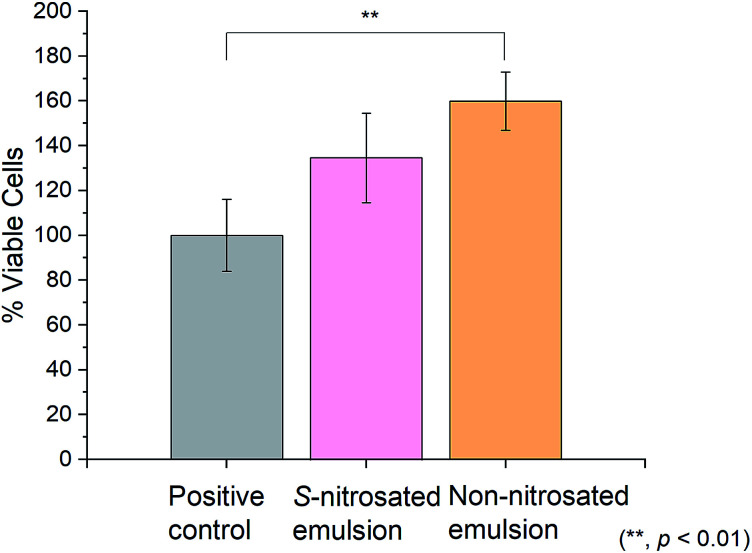
Analysis of human dermal fibroblast cell viability after 24 h of treatment with either an *S*-nitrosated emulsion or a non-nitrosated emulsion in comparison to untreated HDFs (positive control). Each data point represents an average of *n* ≥ 9 replicates and the standard deviation.

Cell viability was assessed using Colorimetric Cell Viability Kit I-WST-8 (CCVK-1) to investigate the potential toxicity of the *S*-nitrosated and non-nitrosated emulsions on HDFs. Since CCVK-1 is a solution that incorporates the electron mediator, 1-methoxy PMS, contact time was minimized and sensitivity was increased in comparison to traditional tetrazolium salts. The results of this assay indicate that neither the *S*-nitrosated nor non-nitrosated emulsion are toxic to HDFs, shown in Fig. 8. Additionally, the consistently lower cell viability of HDFs treated with the *S*-nitrosated emulsion, 135% ± 20% *versus* the non-nitrosated emulsion, 160% ± 13% indicate the possibility of increased cell proliferation using the non-nitrosated emulsion.^52^ The promising results may be due to the fact that the surroundings of the cells consisted, in a greater ratio, of hyaluronic acid and vitamin E in the absence of GSNO.^53,54^ This information suggests that an additive to the base emulsion formulation increases HDF cell viability.^55,56^ Due to the variation in sample homogeneity, further cell studies would be essential before application of this emulsion for antibacterial or cell proliferation purposes.

## 4. Conclusions

The study reports the first preparation of an NO-releasing emulsion with *S*-nitrosothiol groups. The matrix was prepared using a formulation that is intended for topical use and additives such as vitamin E and hyaluronic acid. These incorporated in order to expand the benefits of the emulsion on dermal tissue. GSNO was added to the emulsion to serve as an NO donor at a concentration that allowed for a low NO release. Such release was quantified *via* chemiluminescence-based detection of NO. The targeted NO release was adjusted in order to obtain NO release values that would have specific physiological reactions such as cell proliferation and tissue injury protection. The pH of the emulsion was measured over the course of 12 weeks. A decrease in pH level was observed. Although the pH of the emulsion decreased during the first weeks, it was seen that the pH did not change drastically as measured during the final weeks. Therefore, the ideal storage conditions for this sample involve refrigeration at 4 °C. Using kinematic viscosity, it was determined that the emulsion viscosity is appropriate to be used on delicate areas where external force is not needed or would damage the surrounding tissue. Preliminary cell studies indicate that neither the *S*-nitrosated nor non-nitrosated emulsions are toxic to HDFs. Future studies will include cell assays to provide insight regarding cell proliferation, apoptosis, and morphology. The preparation of an emulsion with beneficial properties to the skin such as the addition of vitamin E and hyaluronic acid with GSNO provide a relevant platform for the treatment of non-healing wounds or ulcers.

## Conflicts of interest

There are no conflicts to declare.

## Supplementary Material

RA-009-C9RA03840J-s001
