# Supplementary material for: Nitric oxide-releasing emulsion with hyaluronic acid and vitamin E
Source: RSC Adv. 2019 Jul 15;9(38):21873–80. doi: 10.1039/c9ra03840j (PMC8009199; doi:10.1039/c9ra03840j)
Supplement: RA-009-C9RA03840J-s001 [file RA-009-C9RA03840J-s001.pdf]

Electronic Supplementary Material (ESI) for RSC Advances.
This journal is © The Royal Society of Chemistry 2019

Electronic Supplementary Information

Nitric Oxide-Releasing Emulsion Enforced with Hyaluronic Acid and Vitamin E

Janet P. Yapor,[a] Jenna L. Gordon,[a] Christina N. Henderson,[b] Melissa M. Reynolds*[ac]

[a] Department of Chemistry, Colorado State University, Fort Collins, CO 80523, USA.

[b] Department of Biochemistry and Molecular Biology, Colorado State University, Fort Collins, CO 80523, USA.

[c] School of Biomedical Engineering, Colorado State University, Fort Collins, CO 80523, USA.

* Corresponding author. E-mail: Melissa.Reynolds@colostate.edu

Table of Contents

Figure S1. Nitric oxide release profiles of replicate experiments (*n* = 3).........................1
Figure S2. Cumulative nitric oxide release profiles of replicate experiments (*n* = 3)........1
Figure S3. Measurement of emulsion pH with respect to GSNO concentration 2
Table S1. Summarized kinematic viscosity and density results.......................................2
Table S2. pH analysis of the *S*-nitrosated emulsion..3

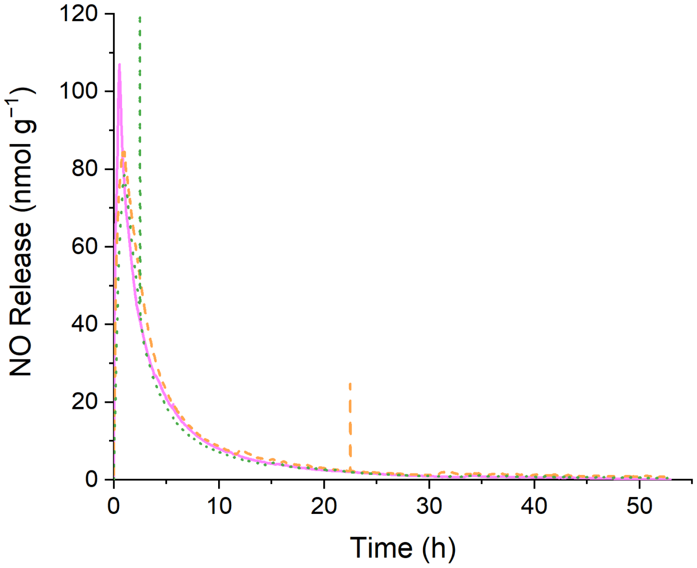

Figure S1. Nitric oxide release profiles of replicate experiments (*n* = 3).

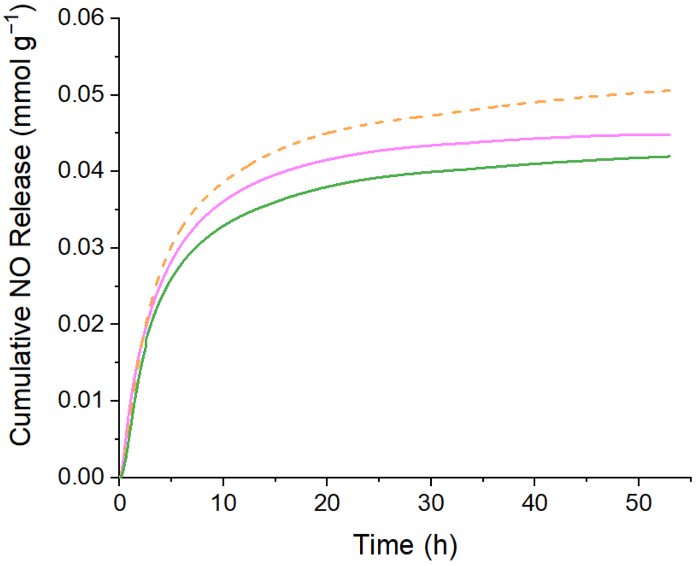

Figure S2. Cumulative nitric oxide release profiles of replicate experiments (*n* = 3).

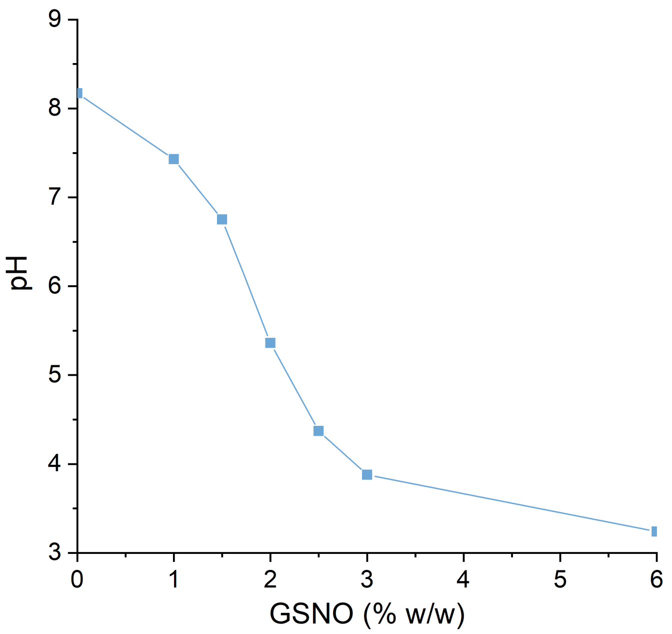

Figure S3. Measurement of emulsion pH with respect to GSNO concentration. The GSNO concentration at 1.72% was determined based on the pH of the emulsion.

Table S1. Summarized kinematic viscosity and density results. All samples were tested in replicate ($n \geq 3$) and the results are reported as the mean ± standard deviation.

	Kinematic Viscosity[a] (cSt)	Density[b] ($g\ mL^{-1}$)
S-nitrosated emulsion	11330 ± 485	0.75 ± 0.05
Non-nitrosated emulsion	9501 ± 1203	0.83 ± 0.06

[a] Values obtained using a Fungilab rotational viscometer.
[b] Values determined experimentally by noting the mass of a specific volume of sample.

Table S2. pH analysis of the *S*-nitrosated emulsion. All samples were tested in replicate ($n \geq 3$) and the results are reported as the mean ± standard deviation.

	pH of *S*-nitrosated emulsion[a]
Week 0	5.5 ± 0.4
Week 1	5.3 ± 0.2
Week 2	4.0 ± 0.1
Week 3	4.0 ± 0.1
Week 4	3.9 ± 0.02
Week 8	3.9 ± 0.01
Week 10	3.9 ± 0.1
Week 12	3.8 ± 0.09

[a] Values obtained using a Mettler Toledo Seven Easy pH meter equipped with a Mettler Toledo InLab® Routine Pro pH probe.
